# Sex-mismatch benefit for speech-in-speech recognition by pediatric and adult cochlear implant users

**DOI:** 10.1121/10.0005806

**Published:** 2021-08-03

**Authors:** Margaret E. Richter, Margaret T. Dillon, Emily Buss, Lori J. Leibold

**Affiliations:** 1 Department of Otolaryngology/Head and Neck Surgery, The University of North Carolina at Chapel Hill, Chapel Hill, North Carolina 27599, USA; 2 Center for Hearing Research, Boys Town National Research Hospital, Omaha, Nebraska 68131, USA margaret_richter@med.unc.edu, mdillon@med.unc.edu, ebuss@med.unc.edu, lori.leibold@boystown.org

## Abstract

This project investigated whether pediatric (5–14 years) and adult (30–60 years) cochlear implant (CI) users benefit from a target/masker sex-mismatch for speech-in-speech recognition. Speech recognition thresholds were estimated in a two-male-talker or a two-female-talker masker. Target and masker speech were either sex-matched or sex-mismatched. For both age groups, performance for sex-matched talkers was worse for male than female speech. Sex-mismatch benefit was observed for the two-male-talker masker, indicating CI users can benefit from a target/masker sex mismatch. No benefit was observed for the two-female-talker masker, suggesting this effect may depend on the relative contributions of energetic and informational masking.

## 1. Introduction

Children and adults spend much of their time in complex, multi-source listening environments that can contain sources producing steady noise (e.g., an air conditioner) and/or sources producing spectro-temporally complex sounds (e.g., people talking). Masking associated with steady noise is thought to reflect degraded representation of the target at the auditory periphery. In contrast, masking exerted by spectro-temporally complex sounds, such as competing speech from a small number of talkers, is often attributed to difficultly segregating and selectively attending to the target. Under these conditions, perceptual similarity and stimulus uncertainty can introduce masking in addition to that associated with the fidelity of cues encoded at the periphery [e.g., Brungart *et al.* (2001), Kidd and Colburn (2017), and Kidd *et al.* (2002)]. These two types of masking are often referred to as informational and energetic, respectively.

Acoustic differences between sounds produced by different sources can support segregation and reduce informational masking. For example, children and adults with normal hearing benefit from a sex mismatch between target and masker talkers (Brungart *et al.*, 2001; Leibold *et al.*, 2018; Stickney *et al.*, 2004), an effect attributed to differences in mean voice pitch and spectral features related to vocal tract length. A sex-mismatch benefit is also observed for children and adults with mild to severe sensorineural hearing loss (Helfer and Freyman, 2008; Leibold *et al.*, 2020), although this effect appears to be smaller than for age-matched listeners with normal hearing.

Cochlear implant (CI) users do not have access to all the cues available to listeners with normal hearing [reviewed by Stickney (2004)]. The relatively small number of frequency bands in a CI reduces spectral resolution, and failure to provide natural fine structure information is thought to limit access to acoustic cues related to voice pitch and intonation (Chatterjee and Peng, 2008; Luo *et al.*, 2012; Stickney *et al.*, 2004). Although the speech information provided by the CI is degraded, it does convey information about talker sex. For example, Skuk *et al.* (2020) reported that CI users can discriminate between male and female voices, relying more on differences in fundamental frequency (F0) than spectral features related to vocal tract length [see also: Fu *et al.* (2005), Gaudrain and Başkent (2018), and Meister *et al.* (2016)]. This finding raises the question of whether CI users can utilize acoustic differences between talkers associated with talker sex to segregate simultaneous target and masker speech.

Previous research on the sex-mismatch benefit for speech-in-speech recognition in CI users has produced mixed results. Some data indicate that CI users can benefit from a sex mismatch between target and masker speech (Cullington and Zeng, 2008; Meister *et al.*, 2020; Willis *et al.*, 2021), although they do not benefit as much as listeners with normal hearing. In contrast, other data indicate no benefit of sex mismatch between target and masker talkers for CI users (El Boghdady *et al.*, 2019; Stickney *et al.*, 2004). Meister *et al.* (2020) suggested that variability in outcomes across studies could be due to differences in the amount of informational masking across samples of target and masker speech. This possibility is consistent with the variability in performance observed across different stimuli for CI users, although this variability is less than observed for listeners with normal hearing (Cullington and Zeng, 2008; Freyman *et al.*, 2007).

A recent study of children with acoustic hearing provides support for the idea that a sex-mismatch benefit is more likely in conditions with greater informational masking in the baseline (sex-matched) condition (Leibold *et al.*, 2020). Children in that study were 7- to 16-year-olds with and without sensorineural hearing loss. All children with hearing loss wore their personal hearing aids during testing. Word recognition performance was compared for target words produced by a male talker and target words produced by a female talker, each tested in two conditions: with a two-male-talker masker and a two-female-talker masker. For both groups, performance was poorer for the male target in the male masker than for the female target in the female masker, and the benefit of a talker sex mismatch was larger for the male masker (and female target) than the female masker (and male target). In other words, the sex-mismatch benefit was larger when baseline performance was poorer.

Leibold *et al.* (2020) also reported supplemental data from adults with normal hearing indicating a similar asymmetry in performance for the two sex-matched conditions, along with evidence that differences in informational masking in the baseline conditions were likely responsible for that effect. Speech recognition for adults was evaluated with and without spatial separation of the target and masker, a method commonly used to estimate effects of energetic masking while minimizing effects of informational masking (Kidd *et al.*, 2016). Spatial separation markedly reduced differences in performance for the two sex-matched conditions (Leibold *et al.*, 2020), a result interpreted as indicating that informational masking played a large role in the differences observed in the baseline condition (in the absence of spatial separation). These findings support the idea that greater informational masking for the male target words in the two-male-talker masker relative to the female target words in the two-female-talker masker was responsible for the larger sex-mismatch benefit observed in the male masker (i.e., for female target words presented in the two-male-talker masker), although it is not clear what stimulus factors are responsible for this difference.

The project described here investigated whether children and adults who use CIs benefit from a talker sex mismatch when listening to speech in a two-talker masker, with a focus on effects related to performance in the baseline (sex-matched) condition and effects of listener age. Stimuli were identical to those used previously by Leibold *et al.* (2020). Testing pediatric and adult CI users with consistent stimuli also supports rigorous evaluation of group differences. We predicted that adults with CIs would benefit from a sex mismatch in the two-male-talker masker, which is associated with higher informational masking in adults with normal hearing. The prediction for children was less clear because early onset of deafness could impact the cues that pediatric CI users rely on when listening to speech. The sex-mismatch benefit could be smaller in pediatric CI users compared to adult CI users, due to reduced familiarity with the acoustic features of speech, or it could be larger, due to early experience with the subtle cues that are provided by CI stimulation.

## 2. Methods

### 2.1 Participants

Two groups of CI users were recruited: 5- to 14-year-olds and adults ≤60 years old. The upper age limit of 14 years for children was selected based on the data of Corbin *et al.* (2016), which indicate that age-related changes in speech-in-speech recognition performance for children with normal hearing continue until 13–14 years of age. The upper age limit of 60 years for adults was selected to limit effects of age-related decline in performance. All participants spoke English as their primary language, and all had a minimum of 6 months of CI listening experience in at least one ear. In addition, children had a negative history of learning, cognitive, or motor delays based on parental report.

Child participants ranged in age from 5.5 to 14.0 years (*M* = 10.0 yr, *SD* = 3.0). They had severe to profound sensorineural hearing loss in both ears; 13 were bilateral CI users, and 5 wore a CI in only one ear. The age of implantation ranged from 1.1 to 14.0 years (*M* = 5.1 yr, *SD* = 4.6). None of the unilateral CI recipients wore a hearing aid in the un-implanted ear. Children were tested immediately following an appointment with their clinical audiologist. Demographic and audiometric information for the child participants is shown in supplemental Tables 1 and 2.^1^

Adult participants ranged in age from 30 to 60 years (*M* = 50.1 yr, *SD* = 9.1). They had severe to profound sensorineural hearing loss in both ears; 7 were bilateral CI users, and 11 wore a CI in only one ear. Although some of the adult unilateral CI users wore a contralateral hearing aid, this hearing aid was removed for testing. Four of the 7 bilaterally implanted adults were tested unilaterally; for these participants, the device that was removed during testing was either associated with <6 months of listening experience (n = 3), or it was an electro-acoustic device (n = 1). Adult CI participants reported peri-lingual and post-lingual deafness. Demographic and audiometric information for adult participants is shown in supplemental Tables 3 and 4.^1^

### 2.2 Stimuli and equipment

The stimuli were those used previously by Leibold *et al.* (2020). Target stimuli were 30 disyllabic English words familiar to children as young as 5 years of age [see Calandruccio *et al.* (2014)]. Those words were recorded from one adult male and one adult female, with mean F0s of 143 and 214 Hz, respectively. The masker was continuous two-talker speech composed of recordings obtained from two adult males or two adult females, each reading different passages from a children's book. Mean F0s for the masker talkers were 144 and 124 Hz (male) or 210 and 170 Hz (female). Different talkers produced the target and masker speech. All six talkers were native speakers of Standard American English.

Recordings were created in a sound-isolated room using a condenser microphone (AKG-C1000S) positioned 6 inches from the talker's mouth. The recordings were amplified (TDT MA3) and digitized (CardDeluxe) using a 44.1 kHz sampling rate (32 bits). Masker speech was edited to eliminate periods of silence >300 ms. Target words and each stream of masker speech were scaled to normalize the root mean square amplitude. Each pair of sex-matched masker speech recordings was then summed, resulting in two-talker maskers that were approximately 2 min 35 s in duration.

A custom matlab script was used to control the selection and presentation of stimuli. Stimuli were resampled at 24.4 kHz, played through a 24-bit digital-to-analog converter (Tucker Davis Technologies, RM1), amplified (Grason-Stadler, GSI-61), and presented via a loudspeaker (Grason-Stadler, GSI High Performance). Participants were tested while seated in a sound-treated booth, facing a loudspeaker that was mounted at a height of 1 m, at a distance of approximately 1 m.

### 2.3 Procedure

Each participant completed testing in either the two-male-talker or the two-female-talker masker. For children, masker assignment was quasi random. For adults, the first nine participants were assigned to the two-male-talker masker, and the next nine participants were assigned to the two-female-talker masker. Testing order for the male and female target speech was randomized for each participant.

Prior to data collection, all participants completed stimulus familiarization in quiet using a picture book with 30 illustrations, one for each target word. Data were collected using a four-alternative, forced-choice (4AFC) paradigm. A target word was selected at random prior to each listening interval,^2^ and three foils were selected at random and without replacement from the remaining 29 stimuli. The four pictures associated with those words were displayed in black and white prior to and during the listening interval. The pictures transitioned to color after the listening interval, indicating that a response was required. Participants specified the word they heard by touching the corresponding image on a handheld touchscreen monitor. After each response, the image associated with the target blinked on and off in isolation to provide visual feedback.

The two-talker masker was presented at 60 dB SPL, and the level of the target words changed adaptively using a 2-down, 1-up rule estimating the speech reception threshold (SRT) associated with 70.7% correct (Levitt, 1971). The starting level of the target words was approximately 10 dB above the expected SRT, based on pilot data and prior data from individual participants. The initial step size was 4 dB; that was reduced to 2 dB after the second track reversal. Eight reversals were obtained for each track. The signal-to-noise ratios (SNRs) at the final six reversals were averaged to estimate the SRT. Participants completed two adaptive tracks for each target condition (male and female), and the final SRT was the average of the two estimates for each condition. Testing was completed in a single session lasting no more than one hour. All procedures were approved by the Institutional Review Board of the University of North Carolina at Chapel Hill.

## 3. Results

**Fig. 1.**
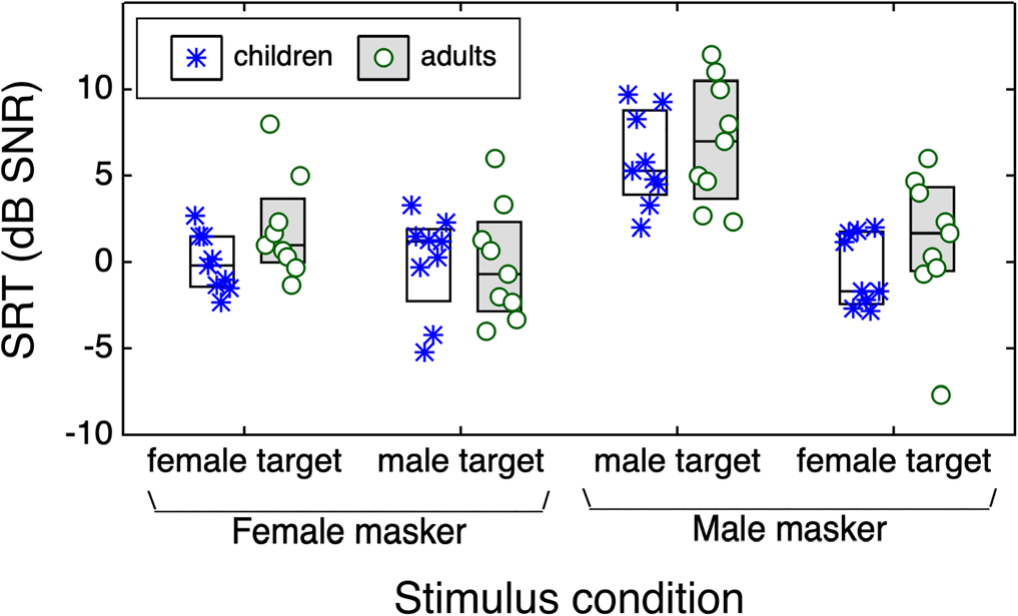
Distribution of SRTs in dB SNR, plotted as a function of masker and target talker sex. Horizontal lines indicate the median, and boxes span the 25th to 75th percentiles. Points indicate data for individual listeners; the order of points for a particular condition and group reflects participant age, from youngest (left) to oldest (right). Symbol and bar style reflect age group, as defined in the legend.

Figure 1 shows estimates of the SRT (in dB SNR) as a function of stimulus condition, indicated on the abscissa. Data are plotted separately for each age group, as indicated by box fill and symbol shape. The points within each box are ordered by listener age, from youngest (left) to oldest (right). The pattern of SRTs across conditions was generally similar for children and adults. For the two-male-talker masker, mean SRTs were higher (indicating poorer performance) for the sex-matched than sex-mismatched condition. In contrast, mean SRTs were similar for male and female targets in the two-female-talker masker. For children, SRTs tended to improve with age. The one-tailed correlation between SRT and child age was significant in the female target and two-female-masker condition *r* = –0.86 (*p* = 0.001). Correlations with age failed to reach significance in the other three conditions (*p ≥ *0.126). There was no evidence of a significant decrement in SRTs with advancing age among adult participants (*r ≤ *0.15; *p *≥ 0.348; one-tailed).

A linear mixed model was used to evaluate the pattern of SRTs across stimulus conditions for children and adults. Fixed effects were age group, masker sex, and sex mismatch, with effect coding for categorical variables. The model included a random intercept for participant. Results are shown in Table 1. There were significant effects of masker sex (*p* = 0.007) and sex mismatch (*p < *0.001), as well as an interaction between masker sex and sex mismatch (*p < *0.001). This interaction reflects the fact that the sex-mismatch benefit differed for the two maskers. Welch's two-sample t-tests indicate that the sex-mismatch benefit was significantly greater than zero for the male masker (*M* = 6.5 dB, *t* = 6.37, *p < *0.001) but not the female masker (*M = *0.6 dB, *t* = 0.64, *p* = 0.527). Lack of a sex-mismatch benefit for the female masker could indicate that performance in the matched-sex condition is largely limited by energetic masking. Neither age group nor any of the interactions with group approached significance (*p ≥ *0.205).

**Table 1.** Comparison of SRTs for adults and children with CIs. Fixed factors and the associated reference conditions were: listener group (adults = 1), masker sex (male = 1), and sex mismatch (mismatch = 1).

	Value	Std. Error	DF	t-value	p-value
(Intercept)	61.91	0.44	32	140.68	<0.001
Group	0.57	0.44	32	1.29	0.205
**Masker**	**1.28**	**0.44**	**32**	**2.90**	**0.007**
**Mismatch**	**−1.77**	**0.23**	**32**	**−7.82**	**<0.001**
Group:Masker	−0.33	0.44	32	−0.76	0.454
Group:Mismatch	−0.19	0.23	32	−0.86	0.398
**Masker:Mismatch**	**−1.46**	**0.23**	**32**	**−6.46**	**<0.001**
Group:Masker:Mismatch	−0.11	0.23	32	−0.48	0.636

## 4. Discussion

The ability to benefit from a sex mismatch between target and masker speech has been consistently observed for children and adults who rely on acoustic hearing (Brungart *et al.*, 2001; Helfer and Freyman, 2008; Leibold *et al.*, 2020), including listeners with hearing loss who wear hearing aids, but results for pediatric and adult CI users have been mixed (Cullington and Zeng, 2008; El Boghdady *et al.*, 2019; Meister *et al.*, 2020; Stickney *et al.*, 2004; Tao *et al.*, 2018; Willis *et al.*, 2021). The present experiment investigated the sex-mismatch benefit for pediatric and adult CI users using a two-male-talker and a two-female-talker masker, stimuli which have been shown to differ with respect to informational masking in normal-hearing adults (Leibold *et al.*, 2020). Whereas prior data utilizing the same stimuli indicate significant sex-mismatch benefit for both maskers in adults with normal hearing and in children with and without sensorineural hearing loss, pediatric and adult CI users tested in the present experiment experienced a significant sex-mismatch benefit only in the presence of the two-male-talker masker.

The discrepancy in sex-mismatch benefit for the two-male-talker and two-female-talker maskers for pediatric and adult CI users is consistent with the idea that stimulus differences are responsible for the discrepancy in results across previous studies of the sex-mismatch benefit in CI users. Different types of masker speech have been utilized in previous studies, from single streams of competing speech to multi-talker maskers (Cullington and Zeng, 2008; Meister *et al.*, 2020; Stickney *et al.*, 2004; Tao *et al.*, 2018). Informational masking can differ markedly across conditions and across stimuli within conditions for listeners with normal hearing (Buss *et al.*, 2021; Freyman *et al.*, 2007) and for CI users (Cullington and Zeng, 2008). It is possible that discrepancies in prior data on the sex-mismatch benefit in CI users may reflect differences in informational masking in the sex-matched baseline condition (Meister *et al.*, 2020). In the present dataset, a sex-mismatch benefit was observed for the masker associated with greater informational masking in adults with normal hearing (Leibold *et al.*, 2020). Although the balance of energetic and informational masking may differ for listeners with normal hearing and CI users, this result is consistent with the idea that the amount of informational masking in the baseline condition impacts the sex-mismatch benefit in both populations [see also: Freyman *et al.* (2008)].

Data reported here for CI users can be compared to those reported previously using these same methods and stimuli for children and adults tested by Leibold *et al.* (2020). For the sex-matched baseline conditions, SRTs for children and adults with CIs were higher for the male speech than the female speech, with mean values of 6.4 and 0.9 dB SNR, respectively. Those SRTs can be compared to values of 0.0 and -3.6 dB SNR for children with normal hearing, and to values of –4.8 and −17.3 dB SNR for adults with normal hearing (Leibold *et al.*, 2020). The absence of a child/adult difference for the CI users is interesting in light of the 13.7-dB age effect observed between children and adults with normal hearing for the female masker. One possible explanation is that children's early CI listening experience is more important than factors related to general maturation. Another possibility is that the cues provided by a CI under these very challenging listening conditions are so impoverished that CI users are not able to segregate and selectively attend to the target regardless of age, such that maturation effects related to these skills do not affect performance. The fact that mean SRTs were at or above 0 dB SNR for the CI participants in the present study provides some support for this idea.

Mean SRTs in the sex-mismatched conditions for pediatric and adult CI users in the current protocol were 0 dB for the two-male-talker and 0.3 dB SNR for the two-female-talker masker, corresponding to sex-mismatch benefits of 6.5 and 0.6 dB, respectively. Those results can be compared to SRTs of −8.9 and –7.1 dB SNR for children with normal hearing, and SRTs of −19.1 and −18.3 dB SNR for adults with normal hearing (Leibold *et al.*, 2020). For children with normal hearing, the sex-mismatch benefit was 8.5 dB for the two-male-talker masker and 3.2 dB for the two-female-talker masker^3^; for adults with normal hearing, the sex-mismatch benefit was 14.3 dB for the two-male-talker masker and 1.0 dB for the two-female-talker masker. Less sex-mismatch benefit for the adults with normal hearing tested with the two-female-talker masker was interpreted as reflecting less informational masking at baseline, reducing the possible range of benefit. A similar explanation might apply to data for pediatric and adult CI users, despite the very poor SRTs in the sex-mismatched conditions. Whereas normal-hearing listeners can benefit from amplitude fluctuations in a speech masker, this benefit is largely absent in CI users (Cullington and Zeng, 2008). The degraded information provided by CI stimulation may limit the benefit derived from cues supporting segregation of competing speech, but that benefit can be observed under some conditions. Interestingly, the sex-mismatch benefit observed by Leibold *et al.* (2020) for children with mild to severe hearing loss who wore hearing aids (4.5 dB, male masker; 1.2 dB, female masker) was similar to that observed for CI users in the present study (6.5 dB, male masker; 0.6 dB, female masker).

Limitations of the present study include the relatively limited sample size, reliance on across-subject comparisons of masker effects, possible differences in intelligibility of the male and female target talkers, and the fact that adults who typically make use of bimodal stimulation were tested with their CI alone, a condition that may be unfamiliar. The upper age limit of 60 years could be problematic when comparing results to published data on younger adults with normal hearing, although this concern is mitigated by the absence of evidence that SRTs increased with age in adult participants in the present dataset. A final consideration is that the relative contributions of energetic and informational masking may differ for CI users and listeners with acoustic hearing, due to differences in the speech cues represented at the periphery in these populations [e.g., Stickney *et al.* (2004)]. The pattern of SRTs across conditions and comparison to data from adults with normal hearing are consistent with greater informational masking with the male masker compared to the female masker, but additional data would be needed to confirm that interpretation.

The current findings may have clinical implications. The ability to recognize speech when several people are talking at the same time poses a substantial challenge to CI users, even when multiple segregation cues are available, such as when talkers are separated in space (Loizou *et al.*, 2009). Understanding the extent to which CIs represent acoustic differences between talkers, and the extent to which listeners take advantage of these differences, is critically important for improving communication outcomes in real-world settings.

## Footnotes

^1^ See supplementary material at https://www.scitation.org/doi/suppl/10.1121/10.0005806 for supplemental Tables 1–4.

^2^ Targets did not repeat within sequential blocks of 30 trials. For example, a track containing 30 trials would contain each target exactly once. The number of trials in each track varied because the stop criterion was based on the number of reversals.

^3^ These values are not the same as those reported by Leibold *et al.* (2020). Sex-mismatch benefit in the current paper is reported for each masker, whereas the previous paper reported benefit for each target.
